# Childhood trauma and malevolent creativity: the mediating role of positive psychological capital and gender differences

**DOI:** 10.1186/s40359-025-03390-9

**Published:** 2025-09-25

**Authors:** Wenfu Li, Mengru Qiu, Jinmei Liu, Aoxue Zhang, Fangfang Xu, Yan Wang

**Affiliations:** 1 School of Rehabilitation Medicine, Jining Medical University, No. 133, Hehua Road, Taibaihu District, Jining, 272067 Shandong Province China; 2 School of Mental Health, Jining Medical University, No. 133, Hehua Road, Taibaihu District, Jining, 272067 Shandong Province China; 3 School of Psychology, Shandong Normal University, Jinan, 250358 China

**Keywords:** Malevolent creativity, Childhood trauma, Psychological capital, Gender difference

## Abstract

**Background:**

Malevolent creativity refers to the creative processes used by an individual to deliberately harm others. Childhood trauma is typically associated with increased malevolent creativity and reduced positive psychological capital. Prior studies have also revealed significant gender differences in malevolent creativity. However, the mediating and moderating effects behind this relationship are still unclear. This study aimed to investigate the mediating effect of psychological capital and the moderating role of gender in the association between childhood trauma and malevolent creativity.

**Method:**

A sample of 1501 college students (mean age = 20.26 years, SD = 1.28; 48.6% women) from China was investigated via questionnaires. All the subjects completed the Short Form of Childhood Trauma Questionnaire (CTQ-SF), the Positive Psychological Capital Questionnaire (PPQ), and the Malevolent Creativity Behavior Scale (MCBS). PROCESS 3.1 was used to test the moderated mediating model.

**Results:**

The results revealed significant gender differences in terms of psychological capital and malevolent creativity, with male students scoring significantly higher than female students. The moderated mediating model analysis revealed that childhood trauma had a positive direct effect on malevolent creativity and that psychological capital mediated this association. Moreover, this mediating model was moderated by gender such that childhood trauma had a stronger influence on malevolent creativity through psychological capital in males than in females.

**Conclusion:**

The present study highlights the gender difference in the mediating role of psychological capital in the association between childhood trauma and malevolent creativity from a positive psychology perspective. The strengths and limitations of the current study are discussed.

## Background

Creativity is widely regarded as the ability to produce original, useful, and valuable ideas or products within a specific cultural setting [1–3]. The term creativity is conventionally associated with positively valued domains, particularly artistic expression, scientific innovation, and technological advancement. However, increasing scholarly attention has been directed toward the dark side of creativity, also known as malevolent creativity, which is defined as the ability to generate novel yet useful ideas to deliberately harm others [4, 5]. Examples of malevolent creative applications include innovative terrorist attacks, terrible crimes, and daily behaviors such as creative mischief, cheating, theft, kidnapping, and sexual harassment [5–7]. Malevolent creative behavior is difficult to prevent once it occurs because it usually causes damage in novel or original ways [8]. Therefore, it is essential to investigate the possible antecedents of malevolent creative behavior, enhance our understanding of this phenomenon, and thereby prevent its occurrence.

Previous studies have systematically investigated both environmental and individual predictors of malevolent creativity [for a meta-analysis, see 9]. Several studies have found that malevolent creativity is associated with individual difference factors including personality traits [9–11], motivation [12], justice sensitivity [13], aggression [14], moral disengagement [15], and anger [16]. Other studies have also identified environmental influences on malevolent creativity. For example, studies show that parental warmth reduces the likelihood of malevolent creativity, whereas parental rejection and overprotection increase it [17, 18]. Several studies also found that situational factors, such as social exclusion, threatening information, parochial altruism, and negative moral emotions, were linked to higher malevolent creativity [19–24].

The Four P’s model (Person, Process, Product, Press) posits that Press (external environmental influences) constitutes a foundational dimension shaping creative outcomes [25]. Additionally, according to hostile attribution bias theory, early environmental factors like adverse childhood experiences makes individuals more likely to perceive ambiguous situations as threatening, which may lead to aggressive or harmful behavior [26]. Childhood trauma is a worldwide phenomenon that strongly predicts adverse behavioral outcomes, such as aggressive behavior [27], non-suicidal self-injury [28], and deception [29]. Given that malevolent creativity inherently involves harmful intentions toward oneself or others [5, 30, 31], it can be hypothesized that childhood trauma may contribute to its development. Recent studies have demonstrated that adverse childhood experiences significantly increase the risk of malevolent creative behavior [11, 14], but the precise mechanisms underlying this association remain poorly understood. Therefore, this study intended to examine how childhood trauma related to malevolent creativity, whether this relationship was mediated by positive psychological capital, and whether gender moderated these effects.

### Childhood trauma and malevolent creativity

Childhood trauma, one of the most common public health problems worldwide, is an adverse experience that occurs in one’s early life before the age of 18 years [32]. Previous studies revealed a significant association between childhood trauma and later behavior problems and mental health outcomes [33–35]. Exposure to childhood trauma has been repeatedly and reliably associated with depression, anxiety, food addiction, aggression, obsessive‒compulsive disorder, and schizotypy [35–40]. Childhood is a critical period for the development of individual creativity [41, 42]. Adverse childhood experiences are often linked to negative parenting styles (e.g., low warmth, high criticism) [43]. These parenting behaviors may subsequently increase the risk of malevolent creativity development [18]. Studies have shown that childhood trauma was negatively associated with general creativity [44] but positively related to malevolent creativity [11] among college students. Recent research has further established childhood trauma as a significant predictor of malevolent creativity [14]. Participants who had experienced four or more adverse childhood events had greater malevolent creativity than did participants who had experienced fewer adverse childhood events [45]. Therefore, on the basis of the literature above, it can be posited that childhood trauma can significantly and positively predicts malevolent creativity. Accordingly, we propose the following hypothesis:

#### H1

Childhood trauma significantly and positively predicts malevolent creativity.

### Positive psychological capital as a mediator

Psychological capital theory posits that psychological capital functions as a protective factor for individuals confronting adverse environments [46]. It is therefore necessary to examine whether psychological capital may mediate the impact of childhood trauma on malevolent creativity. As a positive psychological resource, positive psychological capital has received widespread attention and utilization since the advent of positive psychology [47]. Positive psychological capital is defined as an individual’s positive psychological state characterized by four dimensions: self-efficacy, hope, optimism, and resilience [46]. Previous studies have indicated that positive psychological capital is related to some positive outcomes, such as higher-level well-being, life satisfaction, positive emotion, mental health and academic performance [48–51], which are usually associated with creativity [52–56]. Several researchers have provided direct evidence for the association between positive psychological capital and creativity within the organizational field [57, 58]. Other studies have shown that positive psychological capital is positively related to creativity among college students and adults [59, 60]. Recently, He [61] reported that positive psychological capital was significantly positively associated with multiple measurement approaches to creativity, such as divergent thinking, creative combination and creative problem solving, and Xu [62] reported that positive psychological capital had a positive effect on the level of creativity of college students. Therefore, it is reasonable to expect that positive psychological capital can predict creativity.

Creativity consists of neutral creativity, benevolent creativity, and malevolent creativity [63]. Malevolent creativity is typically considered a creative behavior or idea that is generated to harm others [31]. Previous studies have shown that both anger and aggression are positively associated with malevolent creativity [14, 16]. Other studies have indicated that positive psychological capital is significantly negatively associated with both anger and aggressive behaviors [64, 65]. Therefore, positive psychological capital may help reduce malevolent creativity. More studies have linked psychological capital dimensions, such as resilience, to malevolent creative behaviors [66]. Notably, a recent study has confirmed an inverse correlation between positive psychological capital and malevolent creativity [67]. Thus, based on positive psychology theories and recent evidence, we posit that psychological capital may serve as a negative predictor of malevolent creativity in college students.

Childhood trauma is known to influence many kinds of adult positive traits, including positive psychological capital [68]. Zhang et al. [69] reported that greater childhood trauma was associated with lower positive psychological capital. Other research has revealed that, compared with individuals from families with higher levels of childhood socioeconomic capital, those with lower levels of childhood socioeconomic capital reported poorer positive psychological capital [70]. Liang et al. [71] reported that the positive psychological capital of college students with left-behind experiences in childhood was lower than that of those without left-behind experiences. Numerous studies have reported a significant mediating effect of positive psychological capital on the association between childhood trauma and mental health outcomes such as prosocial behavior and alexithymia [68, 69]. As malevolent creativity can be considered immoral behavior [4, 5], it is plausible to suggest that positive psychological capital can mediate the relationship between childhood trauma and malevolent creativity. Thus, the following hypothesis is proposed:

#### H2

Positive psychological capital mediates the effect of childhood trauma on malevolent creativity.

### Gender as a moderator

The moderating role of gender in the associations among childhood trauma, positive psychological capital, and malevolent creativity may manifest from several angles. First, gender differences in childhood trauma have been widely explored [72, 73]. For example, Chen and Gueta [74] found that females experienced more physical, emotional, and sexual abuse than males. In a national telephone survey, Finkelhor et al. [75] found that the lifetime prevalence of childhood sexual abuse was 26.6% among female adolescents, compared to 5.1% among males. A recent meta-analysis found higher prevalence rates of childhood abuse and neglect among females compared to males.

Additionally, previous studies on gender differences in positive psychological capital have reported inconsistent findings [76, 77]. Several studies have identified gender differences in positive psychological capital [78]. For instance, Zabala-Dominguez et al. [79] found that males scored higher than females on the hope dimension of positive psychological capital. Yu et al. [77] found significantly higher positive psychological capital scores among Chinese male undergraduates compared to their female counterparts. Contrary to these findings, subsequent research has demonstrated significantly higher psychological capital scores in female participants compared to males [80].

Gender differences in malevolent creativity have been investigated in both behavioral and neuroscientific studies. Dumas and Strickland [81] found that males generated significantly more malevolent creative responses to divergent thinking, as measured by alternate-use tasks, than women did. Zhao et al. [82] further found that male students scored significantly higher on malevolent creativity behavior, as measured by a problem-solving task, than female students did. Xu et al. [83] also found that male students scored higher on malevolent creativity, as measured by the Malevolent Creative Behavior Scale [30], than female students did. Other studies using real-world idea generation tasks to measure malevolent creativity found that male participants demonstrated significantly higher levels of malevolent creativity than their female counterparts [5, 16]. In a recent study, Perchtold-Stefan et al. [84] explored gender differences in brain activation via the EEG method while participants generated malevolent creative ideas intended to revenge others and reported that men and women presented different patterns of alpha power changes and functional connectivity changes. While existing studies have consistently demonstrated gender differences in childhood trauma, positive psychological capital, and malevolent creativity, it remains unclear whether gender moderates the relationship between childhood trauma and malevolent creativity among college students.

Previous studies have demonstrated gender’s moderating role in the relationships between childhood trauma, positive psychological capital, and subsequent psychological/behavioral outcomes. For example, Wei et al. [73] analyzed data from 6510 Chinese adolescents and found a stronger association between childhood trauma and depressive symptoms in females than in males. In their study of 2539 Chinese college students, Yan et al. [85] found that adverse childhood experiences significantly predicted lower self-esteem exclusively in female college students. Additionally, Pasha-Zaidi et al. [86] found that gender significantly moderated the childhood trauma-resilience relationship among college students. Moreover, Jia et al. [11] found that childhood neglect more strongly predicted malevolent creativity through Dark Triad traits in male versus female college students. Previous studies have suggested that gender plays a moderating role between childhood trauma and individual factors. However, it remains unclear whether gender moderates the associations among childhood trauma, positive psychological capital, and malevolent creativity.

Therefore, based on previous findings regarding gender differences in childhood trauma, positive psychological capital, and malevolent creativity, as well as gender’s moderating role in the relationships between childhood trauma, psychological capital, and psychological/behavioral variables, we propose the following hypotheses:

#### H3

Gender moderate both the first-stage (childhood trauma → psychological capital) and second-stage (psychological capital → malevolent creativity) pathways of the mediation model, as well as the direct effect of childhood trauma on malevolent creativity.

### The present study

Previous studies have examined the mechanisms linking childhood trauma to malevolent creativity in Chinese college student populations. However, several issues remain poorly understood: First, does psychological capital mediate the association between childhood trauma and undergraduate malevolent creativity? Second, does gender moderate the mediating effects of psychological capital between childhood trauma and malevolent creativity?

**Fig. 1:**
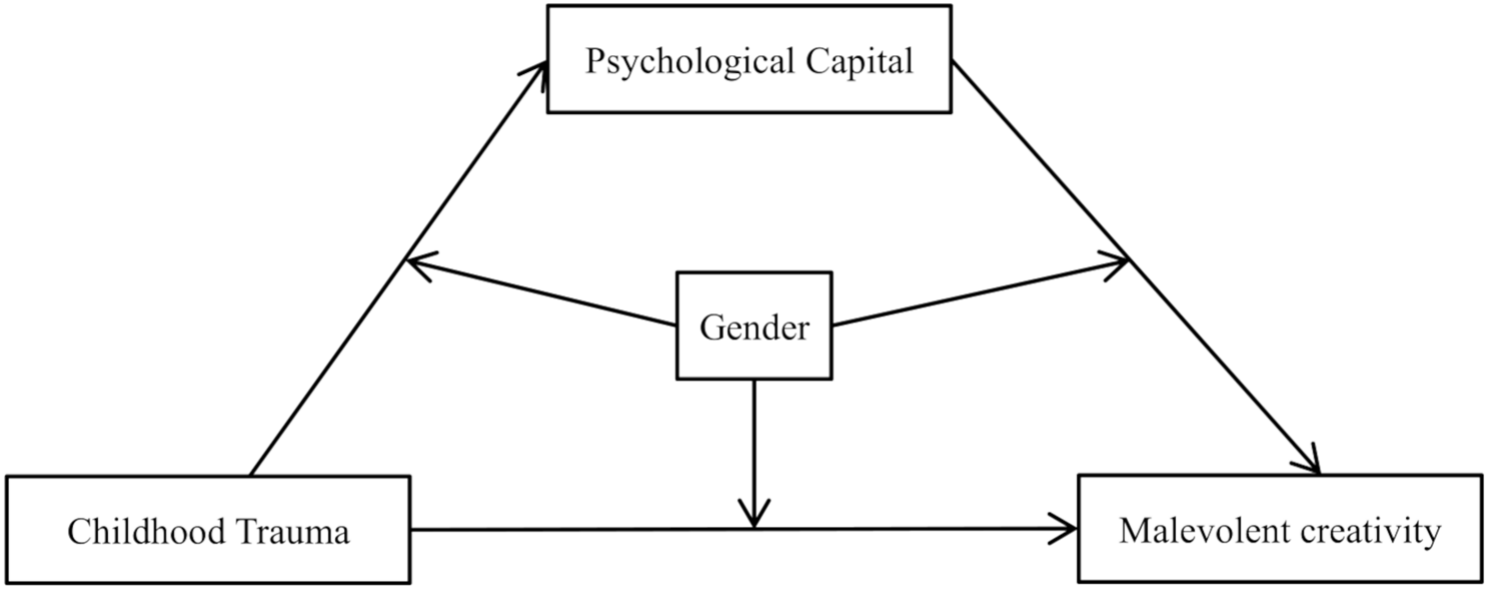
The proposed moderated mediation model of the association between childhood trauma and malevolent creativity

In the present study, we investigated the relationship between childhood trauma exposure and malevolent creative behavior in a sample of Chinese undergraduate students. Based on existing literature, we hypothesized that greater childhood trauma experiences would predict lower levels of psychological capital, which in turn would be associated with reduced malevolent creativity. In other words, childhood trauma may increase malevolent creativity by depleting positive psychological capital. Furthermore, we hypothesized that gender would moderate this mediation effect between childhood trauma and malevolent creativity. Specifically, gender significantly influenced: the association between childhood trauma and psychological capital (first-stage moderation), the link between psychological capital and malevolent creativity (second-stage moderation), and the direct relationship between childhood trauma and malevolent creativity. Figure 1 illustrates the proposed moderated mediation model, specifying: (a) the mediation pathway through psychological capital, and (b) gender’s moderating effects at each stage.

## Method

### Participants

Using a convenience sampling approach, we recruited a sample of 1610 university students from diverse geographical regions in China. All participants volunteered for the online survey (www.wjx.cn) and agreed on the informed consent form. All questionnaires were presented in a concise and easily understandable Chinese version and were either modified or developed following standard procedures. Data from 109 participants were excluded due to invalid or incomplete responses. The final sample comprised 1501 complete and valid questionnaire responses, with an effective recovery rate of 93.23%. Of these, 767 (51.4%) were male undergraduates, and 734 (48.6%) were female undergraduates, with a mean age = 20.26 years and a standard deviation = 1.28. A total of 905 (60.4%) students were only children, and 596 (39.6%) were not only children. A total of 571 (37.9%) of them were from rural areas, and 930 (62.1%) were from towns. The study procedures received ethical approval from the Institutional Review Board of Jining Medical University.

Using G*Power 3.1 software [87], we conducted power analyses for the mediation model and the moderated mediation model. Results indicated excellent statistical power (1.00) for detecting significant effects in our analyses.

### Measures

#### Demographic information

Demographic data including gender, age, birthplace, and sibling status were collected using a custom-designed questionnaire.

#### Short form of the childhood trauma questionnaire (CTQ-SF)

The CTQ-SF was developed originally by Bernstein et al. [32] and is composed of five subscales: physical abuse, emotional abuse, sexual abuse, physical neglect and emotional neglect. The Chinese version of the CTQ-SF, translated and validated by Zhao et al. [88], was used. This questionnaire comprises 25 clinical items and 3 validity items. The participants were asked to assess their objective experiences and subjective assessments during childhood adolescence on a 5-point, Likert-type scale ranging from 1 (“Never true”) to 5 (“Very often true”), such as “When I was growing up, I believe that I was psychologically abused”. This questionnaire has been validated in China and has satisfactory reliability and validity [88]. The total score was computed by summing the responses to 25 clinical items. Higher CTQ-SF scores indicate more severe childhood trauma experiences. The Cronbach’s alpha coefficient of the CTQ-SF in present study was 0.898.

#### Positive psychological capital questionnaire (PPQ)

The PPQ is a 26-item self-reported scale that was developed to assess the reliability and validity of the positive psychological construct [89]. The items on the PPQ asked about opinions on some situations, such as “I always look on the bright side of things”, and were rated on a 7-point Likert scale. Each item has seven options ranging from totally disagree to totally agree. The PPQ comprises four subscales: self-efficacy, hope, resilience, and optimism [89]. Five negatively framed items were reverse scored before the total score was computed. The higher the score of the PPQ is, the greater the level of psychological capital. The Cronbach’s alpha coefficient of the PPQ in current sample was 0.953.

#### Malevolent creativity behavior scale (MCBS)

The MCBS is a 13-item self-reported scale that was developed to provide a reliable and effective measurement tool to assess the malevolent creativity through everyday behaviors [30]. This scale comprises three dimensions: hurting people (e.g., “How often do you have ideas about new ways to punish people”), lying (e.g., “How often do you think of ways to conceal your misdoings from others”), and playing tricks (e.g., “How often do you think of ideas on the margins of rules, when conventional methods do not work”) [30]. The participants were asked to rate their response on a 5-point Likert scale ranging from never to usually. A total score was calculated by summing all 13 items. Higher MCBS scores indicate more malevolent creative behavior in daily life. This scale is widely used to measure malevolent creative behavior among Chinese college students [11, 17, 90]. The Cronbach’s alpha coefficient of the PPQ was 0.830 in current sample.

### Statistical analysis

In the present study, SPSS software was used to conduct descriptive statistical analysis and correlation analysis. PROCESS, an SPSS macro compiled by Hayes [91], was used to test the mediation and moderated mediation model. Model 4 in PROCESS 3.3 [91] was used to examine the mediating effect of psychological capital between childhood trauma and malevolent creativity. Model 59 in PROCESS 3.3 [91] was used to test the moderated mediation model. Demographic variables (gender, age, birthplace, and sibling status) were included as covariates in Models 4 and 59. All continuous variables were standardized (z-scores) prior to computing interaction terms. Using the bootstrap method, we calculated the 95% bias-corrected confidence intervals (CIs) of the above effects. The number of bootstrap samples was 5000. The path coefficient was considered statistically significant if the 95% CI did not contain zero.

## Results

### Common method bias assessment

Using Harman’s single factor test in SPSS, we assessed the possible common method bias of the data from self-reports of participants. An unrotated exploratory factor analysis was conducted on all 67 items from the three measures. The results revealed that 10 principal components were extracted. The first principal component explained 25.69% of the variance, which was lower than the critical value of 40%. This indicated that the common method bias was not a significant problem in the present research.

### Descriptive statistics and correlation analysis

The results of the descriptive statistics (means, standard deviations) and Pearson correlation analysis are displayed in Table 1. Results showed that childhood trauma was positively associated with malevolent creativity and negatively correlated with psychological capital, while psychological capital was negatively associated with malevolent creativity.

**Table 1:** Descriptive statistics and correlation analysis of the study variables

	M	SD	1	2	3
1. Childhood trauma	37.72	10.48	—		
2. Psychological capital	143.95	23.66	-0.47***	—	
3. Malevolent creativity	11.26	6.84	0.43***	-0.35***	—

Note: *M*, Mean. *SD*, standard deviation. ***P < 0.001

### Gender differences in the study variables

Using an independent two-sample *t* test, we examined the sex differences in the study variables (Table 2). The results revealed that there were significant differences in psychological capital and malevolent creativity between male and female students. Both the psychological capital and malevolent creativity of male students were greater than those of female students. Moreover, there was no significant sex difference in childhood trauma.

**Table 2:** Gender differences in the study variables

	Male	Female	t	*P*
1. Childhood trauma	38.14 ± 10.24	37.27 ± 10.71	1.60	0.110
2. Psychological capital	148.16 ± 22.65	139.55 ± 23.91	7.17	< 0.001
3. Malevolent creativity	11.79 ± 6.69	10.72 ± 6.95	3.05	0.002

### Psychological capital as the mediator

To examine the mediating effect of psychological capital between childhood trauma and malevolent creativity, we used PROCESS 3.3 (Model 4) to conduct the mediation analysis. The results are shown in Table 3. The results showed that childhood trauma had a significant positive predictive effect on malevolent creativity (*β* = 0.44, *P* < 0.001) and a significant negative predictive effect on psychological capital (*β* = -0.46, *P* < 0.001). Psychological capital significantly negatively predicts malevolent creativity (*β* = -0.26, *P* < 0.001). The residual direct effect of childhood trauma on malevolent creativity was also significant (*β* = 0.32, *P* < 0.001). These findings indicate that psychological capital has a partial mediating effect on the association between childhood trauma and malevolent creativity. The indirect effect of psychological capital was 0.12 (product of -0.46 and − 0.26), with a 95% CI of [0.092 to 0.150], indicating that the mediating effect of psychological capital was statistically significant. The indirect effect accounted for 27.27% of the total effect.

**Table 3:** The mediating effect of psychological capital

Variable	Model1				Model2				Model3			
	Malevolent creativity				Psychological capital				Malevolent creativity			
	B	SE	β	t	B	SE	β	t	B	SE	β	t
Gender	-0.68	0.33	-0.05	-2.09***	-6.62	1.02	-0.14	-6.51***	-1.18	0.32	-0.09	-3.68***
Age	-0.04	0.12	-0.01	-0.29	0.96	0.39	0.05	2.48*	0.04	0.12	0.01	0.31
Residence	0.56	0.35	0.04	1.59	6.26	1.10	0.13	5.71***	1.03	0.34	0.07	2.99**
Only Child	-0.50	0.35	-0.04	-1.40	-10.41	1.11	-0.22	-9.41***	-1.28	0.35	-0.09	-3.62***
Childhood trauma	0.29	0.02	0.44	18.63***	-1.04	0.05	-0.46	-21.74***	0.21	0.02	0.32	12.11***
Psychological capital									-0.08	0.01	-0.26	-9.39***
*R* ^2^	0.195				0.35				0.24			
*F*	72.62***				157.69***				78.73***			

### Gender as the moderator

**Fig. 2:**
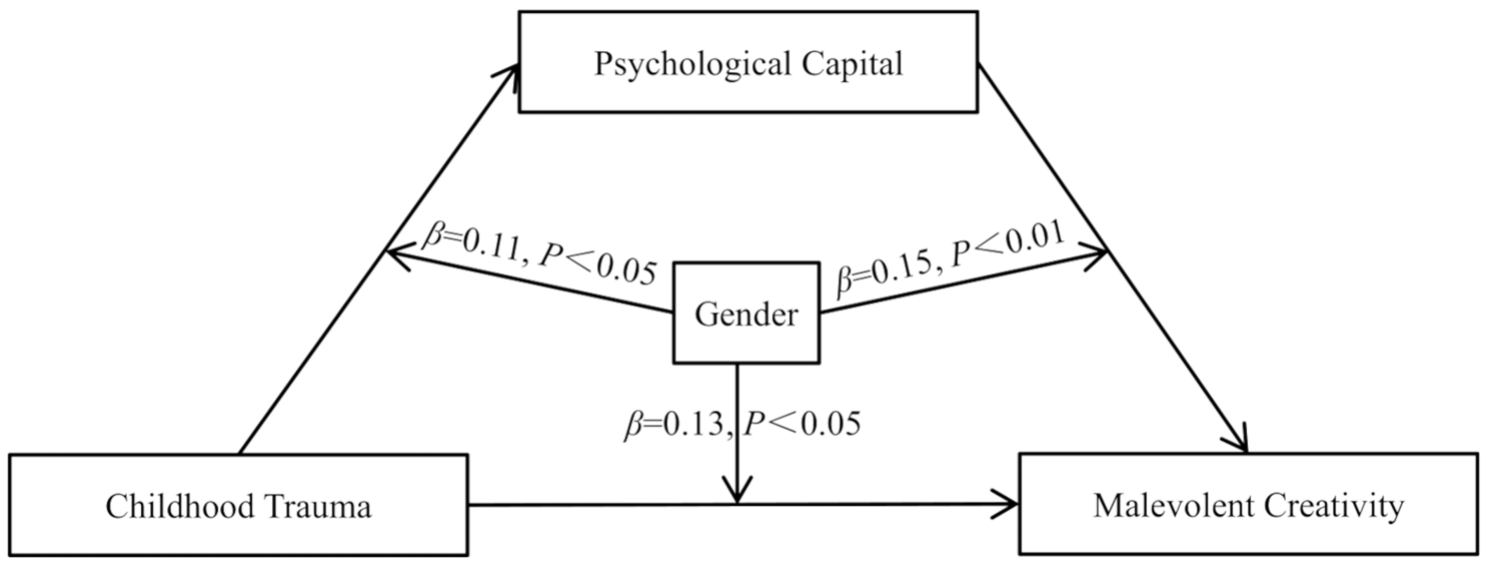
The moderated mediation model of the association between childhood trauma and malevolent creativity

**Fig. 3:**
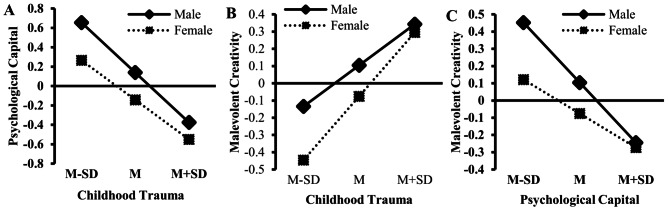
Interaction effects of childhood trauma and gender on psychological capital (**A**) and malevolent creativity (**B**), as well as of psychological capital and gender on malevolent creativity (**C**)

The moderated mediation analysis was conducted using PROCESS 3.3 (Model 59) to examine the moderating effects of gender on the path from childhood trauma to psychological capital and malevolent creativity and the path from psychological capital to malevolent creativity. The results are shown in Table 4; Fig. 2. First, the interaction term of childhood trauma and gender significantly predicted psychological capital (*β* = 0.11, *P* < 0.05). The simple slope analysis revealed that the effect of childhood trauma on psychological capital was stronger for male students (*β* = -0.51, SE = 0.030, *t* = -17.08, *P* < 0.001, 95% CI from − 0.5737 to 0.4555) than for female students (*β* = -0.41, SE = 0.030, *t* = -13.83, *P* < 0.001, 95% CI from − 0.4655 to 0.3498; see Fig. 3A). Second, the interaction term of childhood trauma and gender significantly predicted malevolent creativity (*β* = 0.13, *P* < 0.05). The simple slope analysis revealed that the conditional direct effect of childhood trauma on malevolent creativity was stronger for female students (*β* = 0.37, SE = 0.035, *t* = 10.58, *P* < 0.001, 95% CI from 0.3015 to 0.4388) than for male students (*β* = 0.24, SE = 0.039, *t* = 6.07, *P* < 0.001, 95% CI from 0.1615 to 0.3157; see Fig. 3B). Third, the interaction term of psychological capital and gender significantly predicted malevolent creativity (*β* = 0.15, *P* < 0.01). The simple slope analysis revealed that the effect of psychological capital on malevolent creativity was stronger for male students (*β* = -0.35, SE = 0.041, *t* = -8.53, *P* < 0.001, 95% CI from − 0.4281 to -0.2681) than for female students (*β* = -0.20, SE = 0.036, *t* = -5.43, *P* < 0.001, 95% CI from − 0.2691 to -0.1262; see Fig. 3C).

**Table 4:** The moderated mediation analysis

Variables	Psychological Capital					Malevolent Creativity				
	*β*	SE	*t*	95% CI		*β*	SE	*t*	95% CI	
				LLCI	ULCI				LLCI	ULCI
Intercept	0.61	0.129	4.75***	0.3593	0.8642	0.29	0.140	2.08*	0.0159	0.5639
Age	0.05	0.021	2.32*	0.0075	0.0904	0.005	0.023	0.20	-0.0401	0.0493
Residence	0.26	0.046	5.57***	0.1672	0.3488	0.15	0.050	2.95**	0.0499	0.2475
Only child	-0.44	0.047	-9.32***	-0.5266	-0.3435	-0.18	0.052	-3.40***	-0.2781	-0.0748
Childhood trauma	-0.62	0.067	-9.28***	-0.7530	-0.4903	0.11	0.086	1.25	-0.0614	0.2755
Gender	-0.28	0.043	-6.57***	-0.3659	-0.1977	-0.18	0.047	-3.84***	-0.2718	-0.0879
Childhood trauma*Gender	0.11	0.042	2.54*	0.0245	0.1895	0.13	0.053	2.51*	0.0286	0.2345
Psychological capital						-0.50	0.088	-5.69***	-0.6704	-0.3267
Psychological capital*Gender						0.15	0.053	2.83**	0.0460	0.2550
*R* ^2^	0.348					0.245				
*F*	132.97***					60.54***				

Note: *P < 0.05; **P < 0.01; ***P < 0.001

We further examined the index of moderated mediation to statistically verify the hypothesized conditional indirect effects across gender groups. The results showed that the index of the moderated mediation model was statistically significant (index = -0.099, boot SE = 0.031, 95% boot CI from − 0.1628 to -0.0399). The mediating effect of psychological capital between childhood trauma and malevolent creativity was stronger for male students (effect = 0.18, 95% CI from 0.1315 to 0.2361) than for female students (effect = 0.08, 95% CI from 0.0475 to 0.1170; see Table 5).

**Table 5:** Conditional indirect effects of childhood trauma on malevolent creativity

Gender	Effect	Boot SE	Boot LLCI	Boot ULCI
Male	0.18	0.027	0.1315	0.2361
Female	0.08	0.018	0.0475	0.1170

Note: SE: standard error; LLCI: lower limit confidence interval; ULCI: upper limit confidence interval

## Discussion

The present research investigated the role of childhood trauma on malevolent creativity, as well as its underlying mechanism. Results confirmed both the mediating role of positive psychological capital, and the gender’s moderating effect on this mediation pathway. That is, the mediating effect of psychological capital was significantly stronger in male versus female college students. Furthermore, the interaction of childhood trauma and gender significantly predicted positive psychological capital and malevolent creativity, and the interaction of positive psychological capital and gender significantly predicted malevolent creativity. These findings have important theoretical and practical implications for psychological interventions targeting malevolent creativity in trauma-exposed college students.

### The direct association between childhood trauma and malevolent creativity

As hypothesized, our results revealed a positive association between childhood trauma and malevolent creativity. This finding aligns with evidence linking various childhood adversities (e.g., trauma, neglect, negative parenting style) to increased malevolent creativity [11, 14, 17, 45, 92]. This result also aligned with social learning theory [93], which posits that children exposed to trauma often acquire aggressive or deceptive problem-solving strategies. Additionally, the essential characteristic of malevolent creativity is intentional damage, which is closely correlated with aggression [5, 14]. Similarly, previous studies have indicated that malevolent creativity is positively associated with aggression [30, 90]. Additionally, childhood trauma is positively related to increased aggressive behaviors [94]. Moreover, more childhood trauma is positively related to greater anger [95], which is positively associated with malevolent creativity [16]. Token together, college students with childhood trauma tended to exhibit higher levels of malevolent creativity. Therefore, our findings indicated that fostering positive childhood environments can reduce traumatic experiences, potentially decreasing the likelihood of developing malevolent creative behaviors.

### The mediating role of positive psychological capital

The present study revealed a negative relationship between positive psychological capital and malevolent creativity. This finding was in line with the results of previous studies that showed that resilience and positive emotion, which are considered the key components of psychological capital [46], could negatively predict malevolent creativity [14, 22, 96]. Positive psychological capital can lead to positive outcomes such as higher-level well-being, life satisfaction, positive emotion, mental health and academic performance [48–51]. A study by He [61] showed that positive psychological capital was significantly positively associated with divergent thinking, creative combination and creative problem solving. Malevolent creativity, as the dark side of creativity [97], is creative behavior intended to deliberately harm others [4, 30]. Individuals with high levels of psychological capital always have more positive emotions and less vulnerability [98] and become more resilient, adaptable, and optimistic about the future [99]. Previous studies have shown that positive psychological capital is negatively related to both anger and aggression [64, 65] and that both anger and aggression are positively related to malevolent creativity [14, 16]. This suggested that a higher level of psychological capital was significantly negatively correlated with decreased malevolent creativity. Therefore, according to the results of the positive association between psychological capital and neural creativity [15, 62], our study further indicated that positive psychological capital could not only aid in the development of benevolent creativity but also alleviate malevolent creativity.

Additionally, our results showed that positive psychological capital partially mediated the association between childhood trauma and malevolent creativity. This findings align with psychological capital theory [47], which posits that childhood trauma may deplete an individual’s psychological resources (e.g., hope, resilience), increasing the likelihood of resorting to unconventional and innovative means to achieve goals. Previous studies have indicated that positive psychological capital typically acts as a mediator between early-life childhood trauma and some later health outcomes [68–70]. Another study suggested that psychological capital partially mediated the association between perceived stress and psychological distress [100]. They reported that psychological capital was a positive psychological resource for coping with perceived stress and positively influenced psychological distress among undergraduates and nursing college students [100]. Another study demonstrated that greater psychological capital was correlated with more innovative behavior [101]. Our results indicated that higher childhood trauma was consistently associated with lower levels of hope, dispositional optimism, resilience, and self-efficacy [102–105], which in turn contributed to malevolent creativity [14, 96]. Therefore, we should emphasize fostering children’s psychological capital to help them counteract negative environmental influences and reduce the likelihood of developing malevolent creativity.

### Gender moderated the effect of childhood trauma on malevolent creativity

The present results showed that the direct effect of childhood trauma on malevolent creativity was moderated by gender. The conditional direct effect of childhood trauma on malevolent creativity was stronger for female students than for male students. Our findings provide tentative support for established gender differences in trauma susceptibility, with female participants demonstrating heightened vulnerability to childhood trauma effects compared to males [72, 73]. Compared with males, females usually experience more physical, emotional, and sexual abuse [74]. Experimental studies using classic divergent thinking tests have also demonstrated that male participants generate significantly more malevolent ideas than their female counterparts [81, 106]. However, the findings of the questionnaire studies were mixed: some studies reported higher malevolent creativity scores as measured by the malevolent Creativity Behavior Scale [30] in males [82, 83], whereas other studies reported higher scores in females [107]. Our study further revealed that males showed higher malevolent creativity scores than females when childhood trauma was low, and the scores increased gradually but were not significantly different according to sex when childhood trauma was high. These findings indicated that greater female susceptibility to childhood trauma and a stronger subsequent increase in malevolent creativity compared to males. This indicated we should pay more attention to female college students who’ve experienced childhood trauma, since by helping them develop their psychological strengths, we can decrease the likelihood they’ll apply their malevolent creative behaviors.

Additionally, our results showed that the predictive effect of childhood trauma on psychological capital was also moderated by gender. Specifically, under conditions of low childhood trauma, males exhibited higher levels of positive psychological capital than females, whereas at higher levels of childhood trauma, both groups exhibited lower psychological capital overall. Our findings aligned with the social role theory, which explained these gender differences through socialization processes and the reinforcement of occupational roles [108]. This findings were also confirmed by the results of meta-analytic evidence [109]. Previous study have shown that males generally exhibit higher levels of psychological capital than females, with significant gender differences in how parent-child capital relates to psychological capital outcomes [110]. Another study revealed that females were more easily influenced by childhood adverse experiences [111]. Wei et al. [73] also showed female adolescents developed more depression from childhood trauma than males. Similar results were also found by Prachason et al. [112]. Our findings suggested that female college students are more affected by low-level childhood trauma, while high-level trauma significantly impacts both genders equally. Therefore, this result indicated that educators should devote increased attention to university students with greater childhood trauma exposure, and implement targeted interventions to strengthen their positive psychological capital.

Furthermore, our results also found gender moderated the association between psychological capital and malevolent creativity. More specifically, males were more likely to have malevolent creativity than females when the level of psychological capital was low. In contrast, there was no gender difference in malevolent creativity when the level of psychological capital was high. These findings indicated that psychological capital can play an important role in reducing malevolent creativity at both genders. Previous studies have shown that psychological capital is related to a decrease in several adverse outcomes. For example, a meta-analysis of the last decade of research on psychological capital and depression revealed that psychological capital is negatively correlated with depressive symptoms [113]. Another study also found that individuals with higher levels of psychological capital had lower risks of mental health problems and substance use disorders [114]. Additionally, a body of research have shown that psychological capital is positively associated with prosocial behavior [68] and creativity [61]. Maslakçı et al. [115] further found that the impact of psychological capital on entrepreneurial intention differed between male and female college students. Our results indicated that males exhibit higher malevolent creativity than females at low levels of psychological capital, whereas both genders show comparably low levels when psychological capital is high. Therefore, we should attempt to increase the positive psychological capital of both male and female students to reduce their malevolent creativity.

Finally, our results also showed that gender moderated the mediating role of positive psychological capital in the relationship between childhood trauma and malevolent creativity. Specifically, the mediating effect of positive psychological capital was significantly stronger in male participants than in females. These findings indicated that males’ positive psychological capital is more susceptible to childhood trauma, consequently leading to elevated malevolent creativity. Therefore, we should focus on male college students with childhood trauma experience, implementing psychological interventions to enhance their positive psychological capital and thereby reduce potential malevolent creative behaviors.

### Limitations and future research directions

This study also has several limitations. First, owing to the use of a cross-sectional survey, the present study only revealed the relationships among childhood trauma, psychological capital, and malevolent creativity but could not examine the causal sequence among these variables. To compensate for this deficiency, future studies could adopt longitudinal methods to further confirm the causal connection between childhood trauma and malevolent creativity. Second, the use of college student samples may limit the generalizability of the findings to other populations. Future research should examine these associations in more diverse populations, including varied age groups, educational levels, and socioeconomic backgrounds. Furthermore, this study focused solely on the mediating role of positive psychological capital. Future research could explore other positive psychology aspects, such as positive emotions, self-esteem, self-actualization, interpersonal relationships, and social support,, that can be examined in the future to investigate their effects on the relationship between childhood trauma and malevolent creativity. Finally, while the MCBS is good at measuring malevolent creative behaviors, it may miss some aspects of malevolent creativity. Future studies could assess malevolent creativity using personality traits [9], cognitive styles [116], and divergent thinking [63] tasks to better understand how childhood trauma relates to malevolent creativity.

### Theoretical and practical implications

This study included a sample of 1501 college students and explored the mediating role of positive psychological capital in the association between childhood trauma and malevolent creativity. Moreover, this mediating model was moderated by gender. Specifically, the mediating effect of psychological capital between childhood trauma and malevolent creativity was stronger for males than for females. Based on the findings of this study, we recommend fostering a positive upbringing environment for children to minimize their exposure to childhood trauma, thereby reducing the likelihood of developing malevolent creativity. Additionally, positive psychological capital, as a mediator of childhood trauma on malevolent creativity, can be a vital target of intervention for college students with a history of childhood trauma exposure. With strengthened positive psychological capital, students’ malevolent creativity may be less influenced by childhood trauma. University educators can carry out some intervention measures to increase psychological capital to intervene in and mitigate the malevolent creativity of college students with a history of childhood trauma. Furthermore, the significant gender moderation effects in our study suggest that child-rearing practices should be adapted according to children’s gender to optimally mitigate trauma-related risks for malevolent creativity development.

## Data Availability

The raw data supporting the conclusions of this article will be made available by the corresponding authors upon request, without undue reservation.
